# Effects of a Protocol Combining a Non-Irritating Shampoo and an Adelmidrol-Based Adsorbent Mousse on Seborrhoea and Other Signs and Symptoms Secondary to Canine Atopic Dermatitis: A Multicenter, Open-Label Uncontrolled Clinical Trial

**DOI:** 10.3390/vetsci11060229

**Published:** 2024-05-21

**Authors:** Chiara Noli, Giada Morelli, Maria Federica della Valle, Carlo Schievano, Skinalia Clinical Research Group

**Affiliations:** 1 Servizi Dermatologici Veterinari, Strada Bedale della Ressia 2, 12016 Peveragno, Italy; 2 CeDIS (Science Information and Documentation Center), Innovet Italia Srl, Via Leonardo Da Vinci 3, 35030 Saccolongo, Italy; 3 Innovative Statistical Research Srl, Prato della Valle 24, 35123 Padova, Italy

**Keywords:** Adelmidrol, atopic, dermatitis, dog, mousse, skin, seborrhoea

## Abstract

**Simple Summary:**

Canine atopic dermatitis (cAD) is frequently accompanied by pruritus, skin lesions and seborrheic disorders like greasy skin, excessive scaling and malodour. The variability in cAD manifestations and severity makes patients need individually tailored therapy, in which topical treatments are often recommended. This study evaluated the performance of gentle shampooing and daily application, as a solo or an add-on treatment, of a mousse formulated with Adelmidrol, adsorbent tapioca starch, and a non-prescription antimicrobial complex, on 46 dogs with symptoms secondary to cAD. Dogs were evaluated on days (D)0, D7, D14 and D28. All the investigated parameters (i.e., seborrhoea, pruritus, cutaneous lesions, skin cytological scores) improved overall, according to both veterinarians and owners. Statistically significant improvements were detected by the clinicians after 14 days, and by the caregivers already at D7. In conclusion, the study protocol rapidly and safely improved the clinical condition of cAD-affected dogs.

**Abstract:**

The present study aimed at evaluating the effect of a gentle shampoo and a mousse containing Adelmidrol, tapioca starch and a non-prescription antimicrobial complex on seborrhoea and other clinical signs secondary to canine atopic dermatitis (cAD). Forty-six dogs with cAD-associated seborrhoea and/or pruritus > 4 cm on the pruritus visual analogue scale (P-VAS) and/or bacterial/*Malassezia* overgrowth were enrolled. The mousse was applied twice daily, and dogs were evaluated at days (D)0, 7, 14 and optionally 28, by means of a skin seborrheic index (SSI), P-VAS, cAD lesion index (CADLI), and a semiquantitative cytological score. The mean SSI value improved during the first two weeks (4.1 ± 0.37 to 1.9 ± 0.30; *p* < 0.0001). The mean P-VAS score (cm) decreased from 6.6 ± 0.19 at D0 to 3.8 ± 0.31 at D14 (*p* < 0.0001). The mean CADLI score dropped from 13.7 ± 1.24 to 8.5 ± 1.14 at D14 (*p* < 0.001). The cytological score for bacteria and *Malassezia* decreased from 3.2 ± 0.10 and 3.2 ± 0.11, respectively, to 1.2 ± 0.19 and 1.2 ± 0.24 (*p* < 0.0001). All the investigated signs further improved at D28. Altogether, these observations suggest that the tested protocol might be useful in managing cAD-associated signs.

## 1. Introduction

Canine atopic dermatitis (cAD) is an increasingly widespread, genetically predisposed chronic inflammatory skin disease caused by an abnormal immune response to environmental allergens and cutaneous barrier abnormalities [1,2,3,4,5,6]. Typical manifestations of cAD are pruritus, erythema and predisposition to secondary infections, but also the excessive presence of scales or sebum and unpleasant odours are commonly reported [4]. In fact, secondary keratinization disorders and alterations of the epidermal lipid film may give the skin and coat a greasy (seborrhoea *oleosa*) or dry appearance (seborrhoea *sicca*), respectively. Such disorders may also favour imbalances of the surface microflora, resulting in bacterial and/or yeast (particularly *Malassezia* spp.) overgrowth [7]. Dryness and skin dysbiosis are in turn predisposing factors for the worsening and chronification of both skin inflammation and pruritus. In order to counteract self-perpetuating inflammation, the therapeutic approach to cAD should therefore target many issues concurrently [8].

Several treatments are available for cAD, aiming at limiting skin inflammation and pruritus and/or restoring skin barrier function [1,9]. Numerous studies have demonstrated the value of individual therapies for cAD (e.g., oclacitinib, ciclosporin, lokivetmab, steroids, allergen-specific immunotherapy), while few are those investigating the rates of success of combined treatments [1]. The response to symptomatic therapy for cAD varies widely among patients, with some getting nearly complete relief from a certain treatment regimen while others are not benefiting at all from that same regimen [4]. Since many phenotypes of cAD are observed, the therapeutic choice should be determined depending on the patient’s condition and the severity of the disease [4,9]. Topical therapy is fundamental when treating atopic dogs in this respect, and dedicated guidelines encourage the improvement of skin and coat hygiene and care [1]. In fact, most moderate-to-severe cAD patients benefit from multimodal or combination therapies that include the use of non-irritating shampoos, sprays, ointments, and mousses [4], while mild cAD may be controlled by topical therapy alone. Topical products are used with the goal of keeping skin inflammation and infections better controlled, therefore decreasing or eliminating the need for systemic therapy. Shampoos were so far considered one of the most effective of the topical therapy options, yet their daily use is time-consuming, requires commitment, and eventually requires space and equipment [10,11] and overly frequent shampooing may be drying and irritating [1] due to the harsh effects of surfactants on the lipid composition of the skin barrier [10,12]. Over the past years, manufacturers have been working on easy-to-use formulations [10] in order to facilitate topical therapy by implementing easier protocols and to improve the quality of life of both cAD-affected dogs and their owners [13,14].

There is a great deal of interest in natural compounds with fewer side effects that can reduce or completely eliminate the need for medications used for cAD [9,15]. Also, there is growing evidence about the existence of the cutaneous endocannabinoid system and its involvement in allergic diseases in the canine species [16]. Palmitoylethanolamide (PEA) is a naturally occurring N-acylethanolamine, the parent molecule of bioactive amides named ALIAmides, and one of the most studied components of this system. Recent studies have shown that PEA concentrations and the expression of its receptors in the skin change during cAD [17,18]. Moreover, the administration of PEA was found to provide significant therapeutic benefit in dogs with experimental hypersensitivity [19] and spontaneous atopic dermatitis [20]. An analogue of PEA, Adelmidrol (i.e., the international nonproprietary name of an ethanolamide derivative of azelaic acid), is a topically effective natural substance for inflammatory skin disorders [21]. Adelmidrol is able to down-modulate the inflammatory response in human and animal keratinocytes by increasing their endogenous production of PEA [16,22]. Adelmidrol also acts by down-modulating skin mast cell degranulation [23] and inhibiting the inflammatory response in canine keratinocytes [22]; mast cells and keratinocytes play particularly important roles in cAD due to their critical locations within the skin and their ability to produce a variety of inflammatory cytokines [24]. Adelmidrol is suitable for topical application by virtue of its nature, which is both hydrophilic and lipophilic [23], and its use has been proven to alleviate chronic inflammatory skin conditions in both humans [25] and dogs [23,26].

The aim of the present multicentre, uncontrolled open study was to evaluate the effectiveness of a protocol combining a novel dermatological mousse with a gentle, non-irritating shampoo in dogs affected by seborrhoea and other related clinical signs secondary to cAD. The mousse was formulated to provide soothing, sanitising and adsorbent effects, as it contained ALIAmide Adelmidrol, tapioca starch and a non-prescription antimicrobial complex (i.e., extracts from *E. purpurea*, undecylenic acid and octopirox), with the latter also being used in the shampoo formulation. Tapioca starch was proven to exert adsorbent properties, soaking up excess moisture, oil and dandruff from the skin surface and to control superficial microflora overgrowth in skin folds [27,28]. The extract of *Echinacea purpurea* displayed a sanitising function by hindering bacterial skin penetration [29,30,31], and undecylenic acid was proven effective against fungi and yeasts (e.g., *Microsporum* and *Candida* spp.) [32,33,34]. Octopirox is a piroctone effective against Gram-positive (e.g., methicillin-resistant *Staphylococcus aureus/methicillin-sensitive Staphylococcus aureus*) and Gram-negative bacteria (e.g., *Pseudomonas* spp.), as well as yeasts and fungi (e.g., *Malassezia* and *Microsporum* spp.) [35,36].

## 2. Materials and Methods

### 2.1. Enrollment Criteria

Client-owned dogs of any age, sex, size and breed affected by symptomatically active cAD but otherwise clinically healthy were eligible for inclusion in the study. The diagnosis of cAD was based on Favrot’s criteria [37]. Along with being in good general health, as confirmed by a physical examination by the licensed participating veterinarians, dogs had to fulfil the entry criteria listed in Table 1.

### 2.2. Design of the Study

This study was designed as a prospective, open-label, multi-centre, 14-day clinical trial (D0–D14); according to the caregivers’ availability, the study time could be optionally extended to 28 days (D28).

Dogs were recruited from different veterinary clinics throughout Italy, and the 24 participating clinicians formed the “Skinalia Clinical Research Group”. This research group includes Board Certified veterinary dermatology specialists as well as clinicians with a special interest and experience in dermatology. The study did not need to be assessed for ethical standards under the Italian Minister of Health’s Decree of 12 November 2011 (clinical testing of veterinary drugs), as the study products are licensed for use in Italy and commercially available and were administered following the producer’s indications and guidelines. Owners gave informed written consent for their dogs to participate in the study and were free to withdraw at any time.

All dogs were treated with the study mousse (Retopix^®^ Mousse, Innovet Italia Srl, Saccolongo, Italy). The owners were instructed to apply the mousse twice daily, adjusting the amount to be delivered according to the extension of the affected body part(s), in order to cover the lesions evenly. Its formulation included Adelmidrol, tapioca starch, and a non-prescription antimicrobial complex consisting of extracts from *Echinacea purpurea*, undecylenic acid and octopirox.

The dogs were washed with a shampoo for the gentle cleansing of skin and coat (Redoderm^®^ Shampoo, Innovet Italia Srl, Saccolongo, Italy) at D0 before starting the treatment; the owners were instructed to repeat the shampooing once or twice, up to 48 h prior to the control visit(s). Thereby, the study dogs were bathed with the shampoo on a weekly or fortnightly basis. The shampoo was formulated with a non-irritating gentle cleansing base containing skin barrier restructuring ingredients (i.e., the zinc salt of traumatic acid (Titalin^®^Zn) [38,39] and ceramides [6,40]) and the same non-prescription antimicrobial complex described for the mousse (i.e., extracts from Echinacea purpurea, undecylenic acid and octopirox); Adelmidrol was present only in the mousse and not in the shampoo.2.3. Clinical Assessment and Skin Cytology

The clinicians performed two visits [days 0 (D0) and D14]; a third visit at D28 was optional according to the caregivers’ availability. Also, owners were instructed to fill out an evaluation form at D7. For each dog, all evaluations were performed by the same clinician and the same caregiver at all times.

Seborrhoea was evaluated by both clinicians and dog owners using a modified Skin Seborrhoeic Index (SSI) [41,42]; as illustrated in Table 2, the following parameters were assessed on a 0–3 scale: malodour, scaling, greasiness, extent of the affected area (% body surface affected).

A validated owner-assessed 10 cm Visual Analog Scale (VAS) [43,44] with descriptors was used for assessing pruritus severity. The distance (in cm) from the bottom of the line to the owner’s mark was measured and recorded. Pruritus was then classified as absent (<2 cm), mild (≥2 and ≤4 cm), moderate (>4 and ≤6 cm) or severe (>6 cm), as previously described [20]. Owners performed a pruritus assessment on D0, D7, D14 and D28.

A validated scale known as Canine Atopic Dermatitis Lesion Index (CADLI) [45] was used to assess skin lesion severity on D0, D14 and D28. Briefly, CADLI evaluation considered the most frequently affected body regions in atopic dogs (i.e., head and pinnae, forefeet, hind feet, ventral thorax and axillae, ventral abdomen and inguinal region) and scored two lesion-type subclusters (i.e., “erythema-excoriation-erosion” referred to as CADLI1, and “alopecia-lichenification-hyperpigmentation” referred to as CADLI2) on a six-point ordinal scale (0–5). When bilateral lesions were present, the most severely affected side was considered in the analyses. The total score was obtained by adding the different scores and ranged between 0 and 50; lesions were then classified as in remission (≤5), mild (>5 and ≤7), moderate-to-severe (>7 and ≤23) or severe (>23).

Surface cytology was performed to evaluate the proliferation of bacteria and yeast on the skin surface of the lesions. Acetate tape impressions were performed on D0, D14 and D28 by repeatedly pressing the tape against the affected sites of the skin. The most severe lesion was chosen for cytological sampling, and the same lesion was sampled again in subsequent visits. Tapes were then stained with a modified Wright’s stain (Diff Quick, Dyaset srl, Portomaggiore (FE), Italy), then rinsed under tap water, air-dried and observed at 40–400×. Cytological findings were scored using a validated 0–4 scale [41,46] evaluating semi-quantitatively the presence of bacteria, yeasts and inflammatory cells.

### 2.3. Investigator and Owner Satisfaction

At the end of the study, two distinct satisfaction surveys were conducted among veterinarians and dog owners. Veterinarians were asked for feedback on the control of seborrhoea and clinical signs overall, while owners were interviewed about the features and efficacy of the mousse. All answers were given according to a 4-item non-numeric scale (i.e., “a lot”, “fairly”, “a little”, “in no way”).

### 2.4. Statistical Methods

The response to treatment of the four efficacy outcome measures (SSI, pruritus-VAS, CADLI and cytological score) at the three or four time points (D0, D7, D14, D28) was analysed using a generalised linear mixed model (GLMM) for repeated measures. For each parameter, the analysis was limited to those subjects with active symptoms at enrollment (Table 1). The random effect in the model was animal, while the fixed effects were sex and reproductive status, age, body weight, time, concurrent therapies and the interaction between the latter two.

The *post hoc* Tukey–Kramer analysis was used to compare individual time points, and Fisher’s exact test was used to compare distributions.

The level of significance was set at *p* < 0.05. Data are expressed as mean ± standard error (SE), unless otherwise stated. Data were analysed using SAS v9.4 (SAS Institute, Cary, NC, USA).

## 3. Results

### 3.1. Dog Population

From June to October 2022, 53 dogs were enrolled, three of which were excluded soon after for non-compliance with the protocol, and four were excluded later due to missing or incomplete records. Therefore, the following demographics pertain to 46 dogs, which were visited by 22 clinicians.

Twenty-six dogs were females (57%) and 20 were males (43%); 15 females (33%) and two males were neutered (4%). The mean age was 6 years (range 11 months–14 years), and the mean body weight was 17 kg (range 3–72 kg). Mixed-breed dogs were most represented (*n* = 10), followed by the English Bulldog, French Bouledogue, Jack Russell Terrier, West Highland White Terrier, American Staffordshire Terrier and toy Poodle (*n* = 3 each); all the other breeds (*n* = 14) were represented by one or two subjects.

Most dogs (*n* = 30/46, 65%) completed the study at D28, while the remaining 16 stopped at D14.

### 3.2. Clinical Presentation

In some individuals, cAD was associated with food allergy (*n* = 2), intertrigo (*n* = 2) and otitis (*n* = 1). Twelve dogs (26%) were not receiving any concurrent therapy, and the remaining 34 (74%) had already started a treatment before enrolment. In particular, 20 dogs were receiving oclacitinib, two of which were also administered injectable anti-canine-IL-31 monoclonal antibody (lokivetmab) and prednisolone, respectively; four dogs were receiving ciclosporin (combined with lokivetmab in one case); seven were only administered lokivetmab and three were undergoing allergen-specific immunotherapy. The distribution of concurrent treatments is summarised in Table 3.

**Figure 1:**
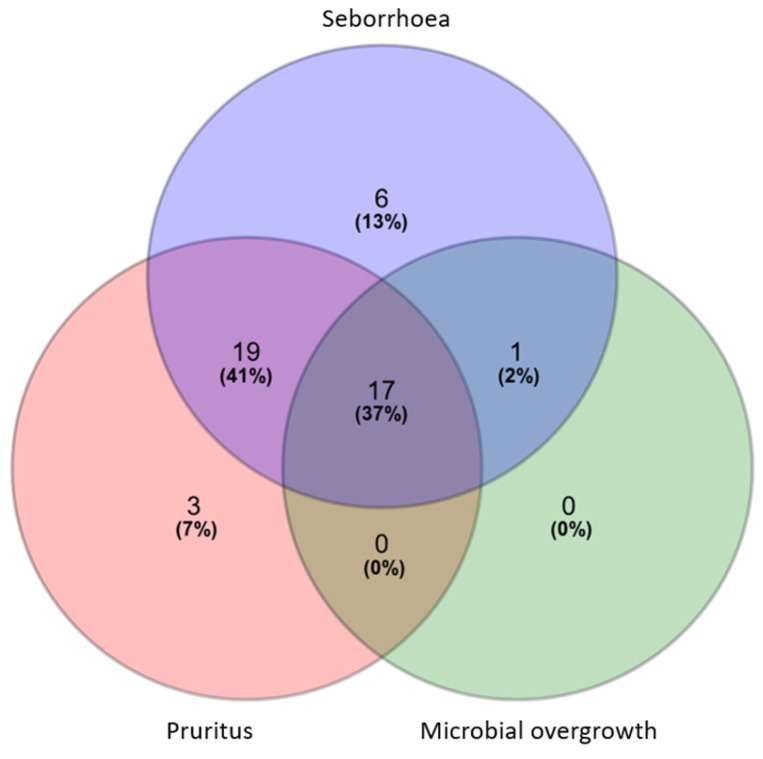
Venn diagram showing the distribution of symptomatically active cAD signs (i.e., seborrhoea, pruritus and microbial overgrowth) at enrolment, expressed as number and percentage of affected dogs (*n* = 46).

Based on the cut-off values at enrolment (Table 1), 17 dogs (37%) showed all clinical signs (i.e., seborrhoea, pruritus and microbial overgrowth); 19 dogs (41%) showed seborrhoea and pruritus, six (13%) showed only seborrhoea, three (7%) only pruritus, and one (2%) both seborrhoea and microbial overgrowth (Figure 1). Overall, 43 dogs (93%) were affected by seborrhoea, 39 (85%) by pruritus, and 18 (39%) by microbial overgrowth.

The raw data are provided in a Supplementary File S1.

#### 3.2.1. Seborrhoea

At D0, seborrhoea type was equally distributed: 15 dogs (33%) had seborrhoea *sicca*, 14 (30%) had seborrhoea *oleosa*, and 14 (30%) had both; 3 dogs (7%) had no seborrhoea.

#### 3.2.2. Pruritus

At D0, 20 dogs (44%) showed moderate pruritus and 19 (41%) showed severe pruritus), while for six (13%) it was mild and for one (2%) it was absent (i.e., <2 cm on VAS).

#### 3.2.3. Skin Lesions

At D0, most dogs (*n* = 27/46, 59%) had moderate-to-severe skin lesions, seven dogs (15%) had mild lesions, six (13%) had severe lesions, and six (13%) had no lesions, according to CADLI scores.

#### 3.2.4. Cytology

At D0, 18 out of 46 animals (39%) entered the study with “positive cytology” (i.e., score of at least 3 points = high or massive number of bacteria/inflammatory cells and/or *Malassezia* spp., identifiable without difficulty). In total, the 18 dogs with evident overgrowth had 31 involved skin areas: hand (23%), abdomen (19%), inguinal (13%), foot (13%), thorax (10%), muzzle (7%), neck (6%), pinna (3%), axilla (3%), and back (3%). Sixteen areas (52%) were positive for bacteria/inflammatory cells, while ten areas (32%) were positive for *Malassezia* spp., and five (16%) for both.

### 3.3. Clinical Evolution

Raw data are provided in a Supplementary File S1.

#### 3.3.1. Seborrhoea

**Figure 2:**
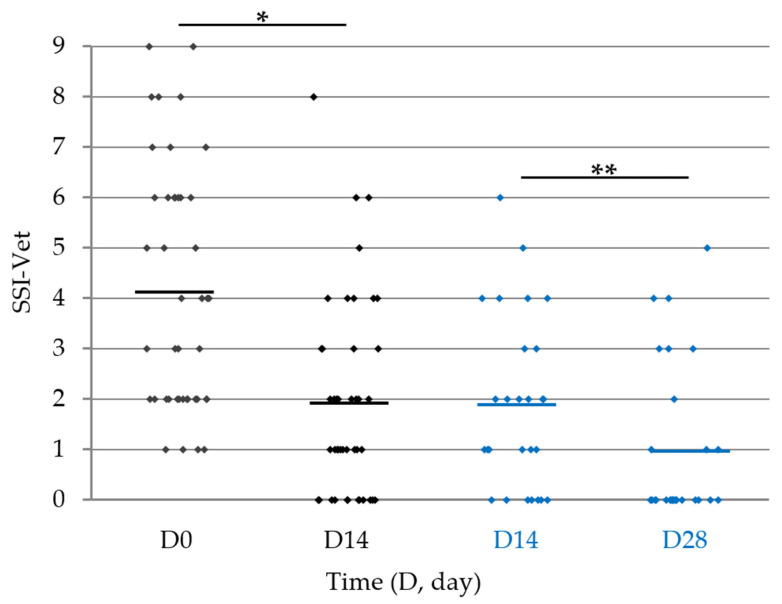
Scatter plot representation of skin seborrheic index (SSI) evaluated by veterinarians in dogs presenting with dry or oily seborrhoea upon entry and ending the study at D14 (*n* = 43/46, black diamonds) or D28 (*n* = 27/43, blue diamonds). The horizontal lines represent the mean. * *p* < 0.0001 (D14 vs. D0); ** *p* = 0.0159 (D28 vs. D14).

The mean SSI value scored by the veterinarians in 43/46 dogs with seborrhoea (Figure 2) improved during the first two weeks (4.1 ± 0.37 to 1.9 ± 0.30; −53%; *p* < 0.0001; *n* = 43) and further decreased in dogs continuing the study until D28 (1.9 ± 0.33 to 1.1 ± 0.32; *p* = 0.0159 vs. D14; *n* = 27). For 67% (*n* = 29/43) of dogs, SSI reduction was at least −50% at D14; such improvement was seen in 80% (*n* = 20/27) of dogs continuing the study until D28.

**Figure 3:**
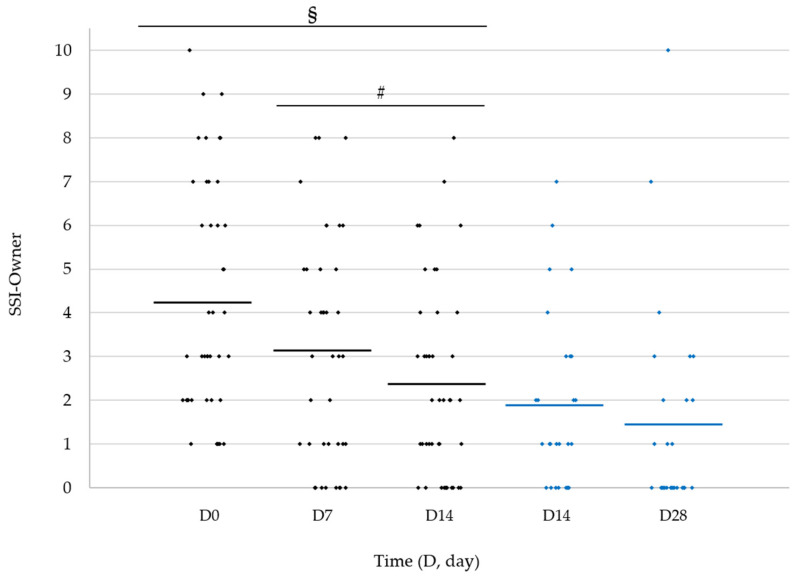
Scatter plot representation of skin seborrheic index (SSI) evaluated by dog owners in dogs presenting with dry or oily seborrhoea upon entry and ending the study at D14 (*n* = 43/46, black diamonds) or D28 (*n* = 27/43, blue diamonds); owners evaluated SSI also at D7. The horizontal lines represent the mean. § *p* = 0.0011 (D14 vs. D0); # *p* = 0.0031 (D14 vs. D7).

Similarly, the mean SSI value scored by the owners (Figure 3) decreased from 4.2 ± 0.41 at D0 to 3.0 ± 0.40 at D7 and was 2.3 ± 0.33 at D14 (*n* = 43); the reduction observed at D14 was significant when compared to both D7 (*p* = 0.0031) and D0 (*p* = 0.0011). Although not significant, a further reduction was seen in dogs continuing the study until D28 (1.4 ± 0.46 versus 1.9 ± 0.39 at D14; *n* = 27).

According to the statistical analyses, dogs’ demographic features (i.e., sex, reproductive status, age, and body weight) and concurrent therapies did not influence the response to the study protocol. In particular, no differences were observed between dogs with and without concurrent treatments with regard to SSI mean values at the study timepoints, nor in the extent of improvement at control visit(s) (Supplementary File S2).

The average malodour score as measured by the veterinarian dropped from 0.9 ± 0.15 (D0) to 0.3 ± 0.09 at D14 (*p* = 0.0017). In the 27 dogs that continued to D28, there was a further (albeit slight and not significant) reduction in the intensity of the malodour (0.2 ± 0.10 at D28). Also based on owners’ evaluation, the reduction in malodour was significant in the first two weeks (1.1 ± 0.16 to 0.5 ± 0.10, *p* = 0.0012). The effect was maintained in the dogs that continued for the next two weeks (0.3 ± 0.12).

At the end of the first two weeks, dry seborrhoea was significantly reduced, according to both veterinarians (−54%, *p* = 0.0002) and owners (−45%, *p* = 0.0046). At the last follow-up visit (D28), the mean scaling score as assessed by the veterinarian was further improved, decreasing significantly from 0.5 ± 0.12 (D14) to 0.2 ± 0.08 at D28 (*p* = 0.0365).

The extent of the body area affected by seborrhoea also tended to reduce throughout the study. In particular, according to the clinicians, the average score decreased from 0.9 ± 0.13 (D0) to 0.5 ± 0.11 (D14) and reached 0.2 ± 0.08 at the end of the study (D28). This trend was significant both in the first two weeks (*p* = 0.0049) and in the last two (*p* = 0.0270), but only for veterinarians and not for owners (*p* > 0.05). In fact, according to caregivers, the average score was 0.9 ± 0.16 at D0, 0.6 ± 0.13 at D7, and 0.5 ± 0.12 at D14, but it reached 0.4 ± 0.13 at the end of the study (D28).

#### 3.3.2. Pruritus

**Figure 4:**
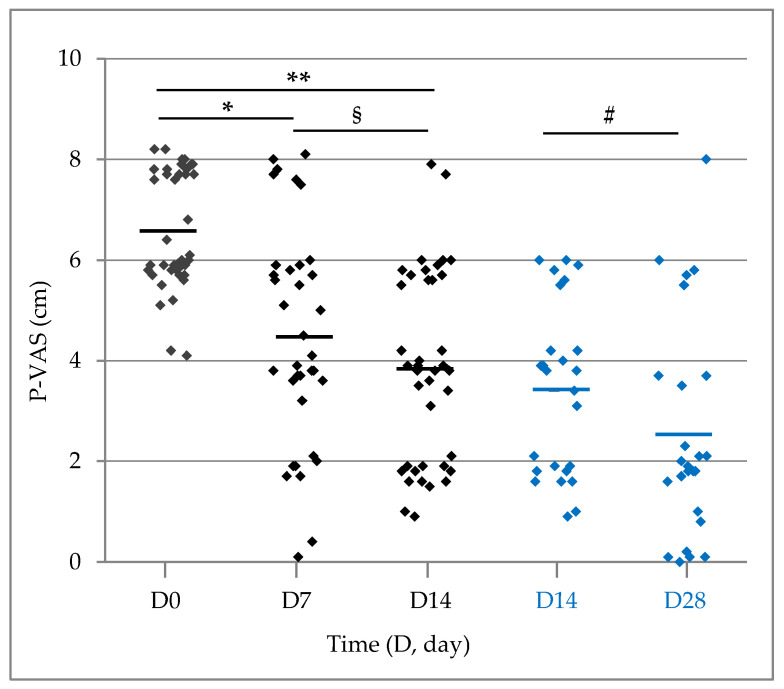
Scatter plot representation of P-VAS score throughout the study in dogs presenting pruritus upon entry and ending the study at D14 (*n* = 39/46, black diamonds) or continuing the study to D28 (*n* = 25/39, blue diamonds). The horizontal lines represent the mean. Thirty-five dogs were evaluated on D7 due to four incomplete reports. * *p* < 0.0001 (D7 vs. D0); ** *p* < 0.0001 (D14 vs. D0); § *p* = 0.0142 (D14 vs. D7); # *p* = 0.0093 (D28 vs. D14).

The severity of pruritus experienced by dogs entering the study with a score greater than 4 (*n* = 39/46; range 4.1–8.2 cm) was significantly reduced at the control visit(s): the mean P-VAS score in cm decreased from 6.6 ± 0.19 at D0 to 3.8 ± 0.31 at D14 (*p* < 0.0001 *n* = 39). For those dogs continuing the study up to D28, P-VAS was 3.4 ± 0.34 at D14 and 2.5 ± 0.44 at D28 (*p* = 0.0093; *n* = 25). Thirty-five owners out of 39 scored pruritus also at D7; the reduction was significant starting from D7 (*p* < 0.0001). Details are given in Figure 4.

As already observed for SSI, co-treatments did not influence the mean P-VAS values at the various observation times (Supplementary File S2).

Compared to the entry value, the P-VAS decreased on average by 2 points (−31%) after the first week and 2.7 points (−42%) after the second study week. Overall, 57% and 77% of the dogs experienced a reduction in pruritus severity of at least 2 points (corresponding to a change to a less severe descriptor), after one and two weeks, respectively (Table 4).

Generally, a reduction in pruritus of at least 50% compared to the entry value is considered clinically significant [44], and this result was achieved by more than a quarter of the evaluated dogs after seven days (*n* = 9/35; 26%) and by 44% after two weeks (*n* = 17/39) (Table 4). It also emerged that 17% and 31% of cases achieved a score less than 2 (i.e., below the normal threshold (Table 4); <2 cm is currently considered the level of pruritus shown by a healthy dog [44]), respectively after one and two weeks. At D28, 76% of dogs showed a reduction in P-VAS score of at least 50%, and over half of the evaluated dogs (*n* = 13/25, 52%) reached a score < 2 cm (Table 4), thus exhibiting a “normal” level of pruritus.

#### 3.3.3. Skin Lesions

**Figure 5:**
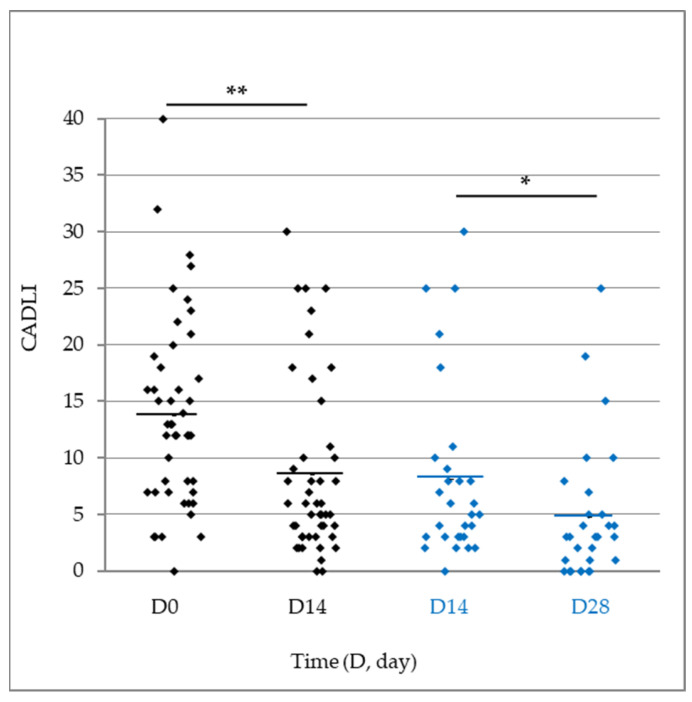
Scatter plot representation of CADLI score throughout the study in dogs ending the study at D14 (*n* = 46/46, black diamonds) or continuing the study to D28 (*n* = 29/46, blue diamonds). The horizontal lines represent the mean. ** *p* < 0.0001 vs. D0; * *p* = 0.0011 vs. D14.

At the beginning of the study, the mean CADLI score was 13.7 ± 1.24 (range 0–40, *n* = 46). After 14 days, the average score dropped significantly to 8.5 ± 1.14 (*p* < 0.0001). In the 29 dogs continuing the study to D28, there was a further significant improvement in the mean lesion score (*p* = 0.0011), which decreased to 4.8 ± 1.12. The analysis of both CADLI sub-scores highlighted similar improvements (Figure 5; Table 5). The improvement in skin lesions was observed regardless concomitant anti-allergic drugs (Supplementary File S2).

Overall, 43% of dogs showed a reduction in CADLI score of at least 50% at D14 (*p* < 0.0001), a percentage that increased to 79% at D28 (*p* = 0.0292). Only six dogs (13%) entered the study with no skin lesions, while 43% (*n* = 20/46) and 76% (*n* = 22/29) were considered to be in remission phase (CADLI score < 5) at D14 and D28, respectively [45].

#### 3.3.4. Cytology

**Figure 6:**
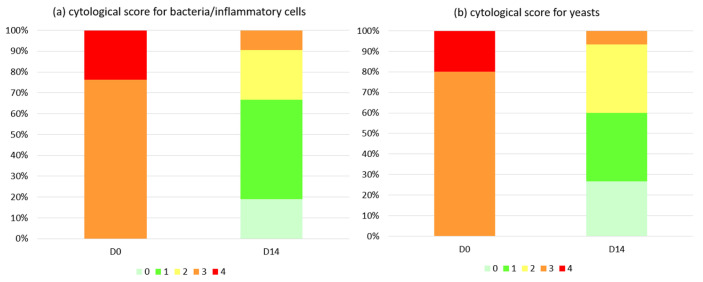
(**a**,**b**) Distribution of severity classes for (**a**) bacterial overgrowth (and/or infiltration of inflammatory cells) and (**b**) *Malassezia* yeasts at day 0 (D0) and after 14 days of treatment (D14). The analysis refers only to dogs that presented scores for bacteria and/or yeasts ≥ 3 upon entry (*n* = 21/46 and *n* = 15/46, respectively). Scores were assigned according to Budach and Mueller [46]: 0 = No bacteria/inflammatory cells/yeast; 1= Occasional bacteria/inflammatory cells/yeast present, but slide must be scanned carefully for detection; 2 = Bacteria/inflammatory cells/yeast present in low numbers, but detectable rapidly without difficulties; 3 = Bacteria/inflammatory cells/yeast present in larger numbers and detectable rapidly without any difficulties; 4 = Massive amounts of bacteria/inflammatory cells/yeast present and detectable rapidly without difficulties.

The cytology score for bacteria/inflammatory cells dropped significantly (*p* < 0.0001) from 3.2 ± 0.10 (D0) to 1.2 ± 0.19 (D14). The cytological score for *Malassezia* showed an almost identical trend, decreasing significantly (*p* < 0.0001) from 3.2 ± 0.11 (D0) to 1.2 ± 0.24 (D14). Figure 6a,b shows the distribution of severity classes for bacterial overgrowth (and/or infiltration of inflammatory cells) and *Malassezia* yeasts. At D28, the cytological score for bacteria was 1 for all 16 samples, and that for *Malassezia* was 1 for 15 out of 16 and 0 for the remaining dog.

### 3.4. Investigator and Owner Satisfaction

After 14 days of treatment, veterinarians declared themselves very or fairly satisfied with the control of signs and symptoms for 91% of their patients; a positive opinion was also expressed for 78% of the patients on the effectiveness of the study mousse in controlling seborrhoea (6% did not respond as they managed the three dogs included without seborrhoea). On the other hand, 83% of the owners affirmed that the study protocol helped improving their animals’ condition.

## 4. Discussion

This study demonstrated the improvement of seborrhoea, microbial overgrowth, pruritus and clinical lesions secondary to cAD following the combination of daily mousse application and bathing with a gentle shampoo on a weekly or every-other-week basis. The study mousse consisted of a novel topical treatment containing Adelmidrol, tapioca starch, and a non-prescription antimicrobial complex (i.e., extracts from *E. purpurea*, undecylenic acid and octopirox) which aimed at soothing the skin [23,25,26], removing the excess oils and flakes [27,28], and balancing the superficial microflora [29,30].

All investigated parameters considered in this trial (i.e., seborrhoea, pruritus, skin lesions, skin cytology) improved overall, with the study products being used either as a sole or add-on treatment. Indeed, it is remarkable that in about ¼ of the enrolled dogs, cAD signs could be managed with our protocol alone. In the remaining ¾ of the dogs, the mousse was applied as an adjunctive measure to ongoing antiallergic systemic drugs, mainly oclacitinib, lokivetmab or immunotherapy, which apparently were not completely successful alone in controlling the symptoms. One may speculate that adding corticosteroids (both oral and topical) could have been helpful to control cAD symptoms in these poorly responding dogs; however, their indiscriminate or chronic use may be associated with side effects, even when using topical products [1,4,9,47,48]. Since cAD is a life-long disease, often with an early onset of clinical signs, the use of safe and easy-to-use interventions, with drug-sparing effects, alone or in combination with systemic antiallergic treatments, is increasingly taken into consideration by researchers and practitioners as an alternative to the use of corticosteroids [9].

Bathing with non-irritating shampoos is an important component of the chronic management of cAD, as it removes debris, scales, grease and allergens, and also provides temporary relief to pruritic dogs [1,11,49]. To what extent the observed effect is due to one or the other product is not easy to establish in the current study, although dogs were not supposed to be washed for at least two days before the visit. New studies testing the mousse alone, shampooing alone, or a comparison between the two are desirable; however, previous literature showed that the effect of shampoos was modest and short-lived, while it could be extended by combining them with foam preparations [11].

Pruritus is a major clinical sign in cAD [1,9,50], and interrupting the itch-scratch cycle is of primary concern, as constant scratching of the skin exacerbates inflammation, worsens skin lesions and may predispose to microbial overgrowth. Also, addressing pruritus is of primary concern to the caregivers, who are often distressed by the pet’s discomfort [14,51].

The success rate in this study was high, as 77% of dogs showed a >2 cm reduction in the pruritus score from baseline (shifting to a lower severity class) after two weeks of treatment. Moreover, 44% and 61% of dogs showed a reduction in pruritus of at least 50% after two and four weeks. Similar improvement rates were obtained by commonly used drugs (e.g., ciclosporin, prednisolone, oclacitinib), although they were associated with greater side effects [52,53,54,55,56,57,58]. The mean reduction in pruritus observed in this study after fourteen days (42%) was higher than those following the application of two commercially available dermatological mousses tested as monotherapy on eight atopic dogs in a previous study (26% and 33% for Foam A and Foam B, respectively) [59]; however, unlike our study, concomitant medications, including shampoos were not allowed in such a protocol.

It is worth mentioning that among the subjects showing P-VAS values over 4 cm at enrolment, 18 were already receiving an antipruritic drug for at least four weeks; the application of the study products as a single or add-on treatment managed to reduce pruritus in 16 of those, an important proof of their add-on, drug-sparing property.

This significant antipruritic action could be explained by improved coat hygiene [1,60] and by increased cutaneous levels of PEA and a reduced inflammation provided by Adelmidrol, as already demonstrated in vitro and in vivo [22,23,25,26].

Almost eighty percent of dogs had lesion scores improved by ≥50% by the end of the study (D28). Like for pruritus, these improvement rates are similar to those obtained by commonly used drugs (e.g., ciclosporin, prednisolone, oclacitinib), sometimes with longer study times (i.e., six to twenty-four weeks) [52,53,54,55,56,57,58,61]. The mean reduction in total CADLI observed in this study after fourteen days (38%) was in line with those following the application of two commercially available dermatological mousses tested as monotherapy on eight atopic dogs in a previous study (38% and 42% for Foam A and Foam B, respectively) [59].

Importantly, three in four dogs finishing the study at D28 had a CADLI score below 5, which is the severity threshold consistent with clinical remission. A similar success rate was also obtained with the oral supplementation of ultramicronized PEA [20].

Together with pruritus and dermatological lesions, seborrhoea is a frequently encountered condition in dogs affected by allergic dermatitis [50]. In the current trial, all criteria related to seborrhoea reduced consistently with time, according to both clinicians and caregivers, the latter noticing an improvement already after one week (although not statistically significant). Among the main features of seborrhoea, malodour and scaling were judged more severe by the owners at D0 and then recorded the highest reduction during the study. This is in agreement with another study testing a topical protocol against greasy seborrhoea, as malodour seems to be the main complaint by the owners when keratinisation disorders occur, and one of the major reasons for shampooing their animals [62]. Also, shampooing probably helped remove some of the scales mechanically [10]. On the contrary, veterinarians scored higher points for evaluating the extent of the affected area, maybe because owners are less trained to quantify.

In a recent work [10], a keratomodulating mousse containing plant extracts (*Ophiopogon japonicus* and *Punica granatum*) proved capable of improving the clinical condition of seborrhoeic dogs; its application, once every two or three days for 24 days, decreased the scores of the same seborrhoea-related parameters investigated in this study (i.e., malodour, scaling, greasiness, and extension) in twelve untreated dogs affected by chronic, primary greasy keratinisation disorder.

Patients with cAD are strongly predisposed to secondary bacterial (most often *Staphylococcus pseudintermedius*) and/or yeast (*Malassezia* spp.) skin and ear infections [1,4,6,7,50]. The assumption is that in some of these patients, the skin barrier defects caused by inflammation and scratching favour microbial infections, which can greatly or completely improve when the inflammatory insult is eliminated [4]. Furthermore, avoidance of repeated systemic and topical antibiotic and/or antimycotic use is recommended to decrease the occurrence of antimicrobial resistance by cutaneous and intestinal microorganisms, and topical use of non-prescription sanitizing properties is preferred.

Based on the above, the significant improvement in the cytological scores of both bacteria and yeast observed after two weeks could be the result of the soothing and sanitising properties of the products used in the current study, empowering the possibilities of decreasing antimicrobial use in cAD and hopefully resulting in less resistance [1,4].

The tested mousse received good to excellent feedback, as owners appreciated the texture and the easy-to-use formulation (data not reported); in recent years, mousse formulations have been proposed in the management of cAD as its application is simple (through massage), it does not require prior wetting of the skin, nor rinsing or drying afterwards, thus allowing direct skin contact and prolonged action [10,59]. Rapid and easily reachable improvements in skin appearance are important features that encourage owner compliance [10].

The present study has some limitations that need to be addressed. First of all, the study had an open-label, uncontrolled and multicentre design. Therefore, the experimental design did not allow for the recognition of a placebo effect in either the clinical evaluator or pet owner, as no control groups receiving a control vehicle mousse daily or just a shampoo were established. Also, having numerous investigators may potentially impact the homogeneity of treatment evaluation; such a feature, which reflects the real field conditions, was chosen to allow the recruitment of a sufficient number of subjects to satisfy the trial objectives within a reasonable timeframe. Second, the study dogs were heterogenous in many features and in therapeutical regimes; however, as mentioned above, this study was conducted under field conditions: all dogs were client-owned, and the role of domestic management and environmental variability on individual cAD manifestation should not be overlooked. Lastly, a larger sample size may have reduced the variability induced by the multi-centre nature of the study.

## 5. Conclusions

Along with weekly or fortnightly gentle shampooing, the daily application of a mousse containing Adelmidrol, adsorbent tapioca starch, and a non-prescription antimicrobial complex, either used singly or in combination with other therapeutic approaches, was found to be an effective topical intervention to reduce seborrhoea secondary to cAD and associated clinical signs. Although future controlled trials are warranted, marked improvements at both the veterinarian and owner assessment were observed in skin signs and symptoms (i.e., seborrhoea, pruritus, skin lesions) and microbial overgrowth following a short course of the study protocol.

## Figures and Tables

**Table 1:** Enrollment criteria.

Inclusion Criteria	Exclusion Criteria
Scaling or greasiness score (assessed by the clinician) > 1, and/or pruritus VAS score > 4 cm, and/or cytology score ≥ 3 [details on the grading scales are reported in Section 2.3 If already provided, maintaining anti-allergic treatments on a stable dose for ≥1 month (oclacitinib, ciclosporin), ≥2 months (monoclonal antibodies, e.g., lokivetmab), or ≥12 months (immunotherapy) Maintaining the same diet and environmental conditions before entering and throughout the study period	established flea allergy dermatitis diagnosis pregnancy and lactation parasitic or dermatophytic infestation, yeast or bacterial skin infections requiring systemic antimicrobial treatment need of oral glucocorticoids application of any topical product * (i.e., creams, shampoos, sprays) in the week preceding inclusion

VAS, Visual Analog Scale. * Except for antiparasitic prophylaxis and the study shampoo.

**Table 2:** Grading scale used to evaluate the Skin Seborrhoeic Index (SSI). The SSI was obtained by summing the four values.

Parameter	0	1	2	3
Malodour	absent	perceptible in proximity to the animal	perceptible at some distance from the animal	strong even at some distance
Scaling	absent	mild	moderate	severe
Greasiness	absent	mild	moderate	severe
Extent (% of the body surface)	<20%	20–50%	50–75%	>75%

**Table 3:** Distribution of concurrent treatment received by the study dogs at enrolment.

					Allergen-specific immuno-	
				Injectable	therapy	
			Oral	lokivetmab	3	
			treatment	7	-	
Ciclosporin			3	1	-	
Oclacitinib			18	1	-	
Oclacitinib + Prednisolone			1	-	-	
*Total dogs with concurrent therapy*			22	9	3	34/46 (74%)

**Table 4:** Treatment success rates for pruritus according to owner-assessed 10 cm Visual Analog Scale (VAS).

	D7	D14	D28
	*n* = 35	*n* = 39	*n* = 25
VAS reduction ≥ 2 cm *p* (vs. previous study time)	20 (57%) <0.0001	30 (77%) 0.0853	23 (92%) 0.6671
VAS reduction ≥ 50% *p* (vs. previous study time)	9 (26%) 0.0006	17 (44%) 0.1448	19 (76%) 0.1398
VAS < 2 cm (“normal”) *p* (vs. previous study time)	6 (17%) 0.0088	12 (31%) 0.1893	13 (52%) 0.3931

**Table 5:** Mean change in lesion severity as assessed by the clinician on the total Canine Atopic Dermatitis Lesion Index (CADLI tot) and the two CADLI subclusters (i.e., CADLI 1 = erythema-excoriation-erosion and CADLI 2 = alopecia-lichenification-hyperpigmentation).

		D0	D14	*p*	D14	D28	*p*
		*n =* 46	*n =* 46	D14 vs. D0	*n =* 29	*n =* 29	D28 vs. D14
CADLI (tot)	Mean SE	13.7 1.24	8.5 1.14	*p* < 0.0001	8.5 1.46	4.8 1.12	*p* = 0.0011
CADLI 1	Mean SE	8.5 0.71	5.2 0.62	*p* < 0.0001	5.2 0.83	3.3 0.96	*p* = 0.0494
CADLI 2	Mean SE	5.2 0.74	3.3 0.64	*p* = 0.0008	3.3 0.75	1.4 0.36	*p* = 0.0417

## Data Availability

The data presented in this study are available in the Supplementary Material.

## Supplementary Materials

The following supporting information can be downloaded at: https://www.mdpi.com/article/10.3390/vetsci11060229/s1. Supplementary File S1: Raw data collected in this study. Supplementary File S2: Figures comparing SSI, P-VAS and CADLI between dogs with and without concurrent treatments.
